# Hydrogen–deuterium exchange in imidazole as a tool for studying histidine phosphorylation

**DOI:** 10.1007/s00216-014-8218-5

**Published:** 2014-10-30

**Authors:** Małgorzata Cebo, Martyna Kielmas, Justyna Adamczyk, Marek Cebrat, Zbigniew Szewczuk, Piotr Stefanowicz

**Affiliations:** Faculty of Chemistry, University of Wrocław, Joliot-Curie 14, 50-137 Wrocław, Poland

**Keywords:** Phosphohistidine, Hydrogen–deuterium exchange (HDX) reaction, Deuterium–hydrogen exchange (DHX) reaction, Mass spectrometry

## Abstract

Isotope exchange at the histidine C_2_ atom of imidazole in D_2_O solution is well known to occur at a significantly slower rate than the exchange of amide protons. Analysis of the kinetics of this isotope-exchange reaction is proposed herein as a method of detecting histidine phosphorylation. This modification of His-containing peptides is challenging to pinpoint because of its instability under acidic conditions as well as during CID-MS analysis. In this work, we investigated the effect of phosphorylation of the histidine side chain in peptides on deuterium–hydrogen exchange (DHX) in the imidazole. The results demonstrate that phosphorylation dramatically slows the rate of the DHX reaction. This phenomenon can be applied to detect phosphorylation of peptides at the histidine residue (e.g., in enzymatic digests). We also found that the influence of the peptide sequence on the exchange kinetics is relatively small. A CID fragmentation experiment revealed that there was no detectable hydrogen scrambling in peptides deuterated at C_2_ of the imidazole ring. Therefore, MS/MS can be used to directly identify the locations of deuterium ions incorporated into peptides containing multiple histidine moieties.

**Electronic supplementary material:**

The online version of this article (doi:10.1007/s00216-014-8218-5) contains supplementary material, which is available to authorized users.

## Introduction

**Fig. 1:**
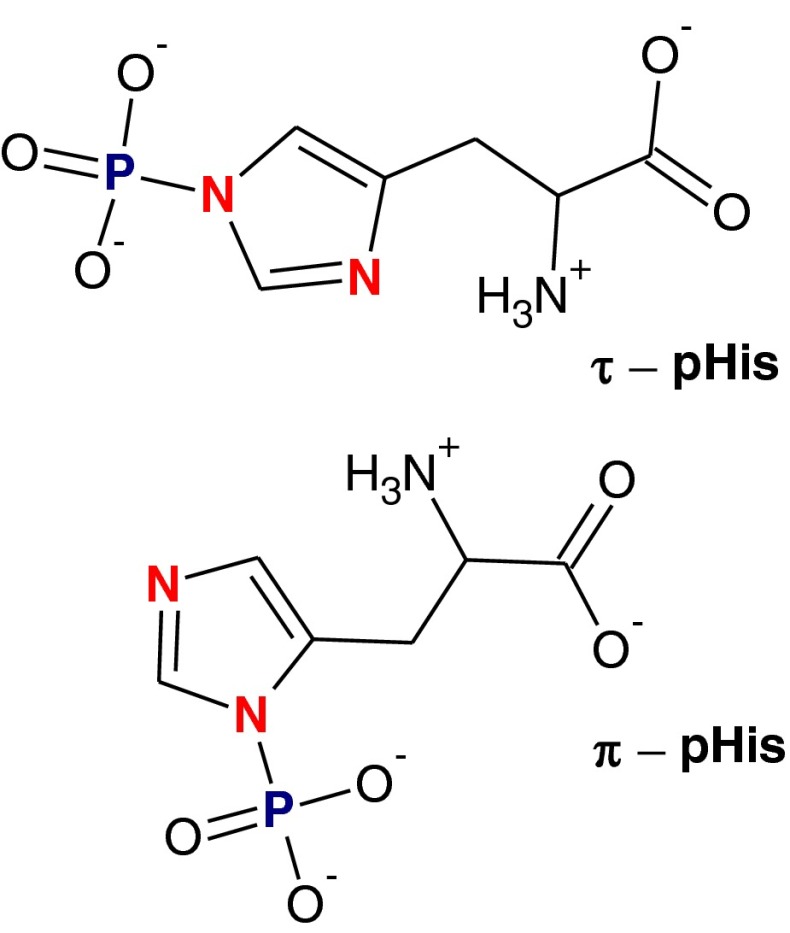
Isomeric forms of phosphohistidine

Phosphorylation is a common posttranslational modification (PTM). The basic amino acids histidine, lysine, and arginine all undergo* N*-phosphorylation. It has been estimated that in eukaryotic cells up to 6 % of the protein phosphorylation occurs at histidine [1]. Phosphohistidine (pHis) exists in two isomeric forms: τ(tele)-phosphohistidine and π(pros)-phosphohistidine (Fig. 1) [2], which are the perfect carriers of the phosphoryl group in biological systems because of the high Δ*G*° for hydrolysis of the P–N bond [3, 4]. It has been postulated that histidine phosphorylation is an essential process in the cellular signaling of bacteria, fungi, and plants [5, 6], and it has also been reported to occur in mammalian signaling pathways [7–10] and to be involved in certain human diseases [11, 12].

The phosphoramidate nitrogen of pHis is not basic, as protonation of this nitrogen would disturb the aromaticity of the imidazole ring. However, the second nitrogen atom in the phosphoimidazole ring* is* relatively basic (its p*K*_a_ is ~7). Protonation of this atom reduces the electron density on the phosphoramidate nitrogen and consequently increases the leaving-group ability of the protonated imidazole ring [4]. Therefore, pHis is unstable under acidic conditions (e.g., in 1 M HCl at 49 °C), and π(pros)-phosphohistidine has a half-life of 18 s while τ(tele)-phosphohistidine has a half-life of 25 s [13].

Due to the acid lability of N-phosphorylated histidine, direct detection of this posttranslational modification in proteins is still a challenge. Even though a few techniques have been applied for this purpose, none of them is considered a general method. The low abundance of pHis peptides in proteomic samples has motivated the development of methods of enriching pHis [14]. Kleinnijenhuis et al. utilized a 10-min gradient with an acidic mobile phase (0.5 % acetic acid) to achieve the HPLC separation of pHis peptides [15]. Hohenester et al. reported the use of 0.1 % formic acid in the analysis of peptides with pHis [16]. Moreover, electron-based fragmentation techniques have been found by Kleinnijenhuis et al. to be useful for analyzing this labile peptide modification [15]. These fragmentation techniques provide more detailed information when determining the locations of phosphorylated histidine sites than CID does. Similarly, phospholysine is also difficult to detect. In previous research, we found the ECD method to be useful for locating sites that have been modified in unstable lysine-phosphorylated peptides due to a significant reduction in neutral losses [17]. On the other hand, reactions of phosphorylating agents (e.g., toxic organophosphorus compounds such as pesticides and nerve agents) with lysine moieties in proteins result in the formation of relatively stable derivatives that are phosphorylated on lysine residues; these can be directly analyzed using mass spectrometric techniques [18].

**Fig. 2:**
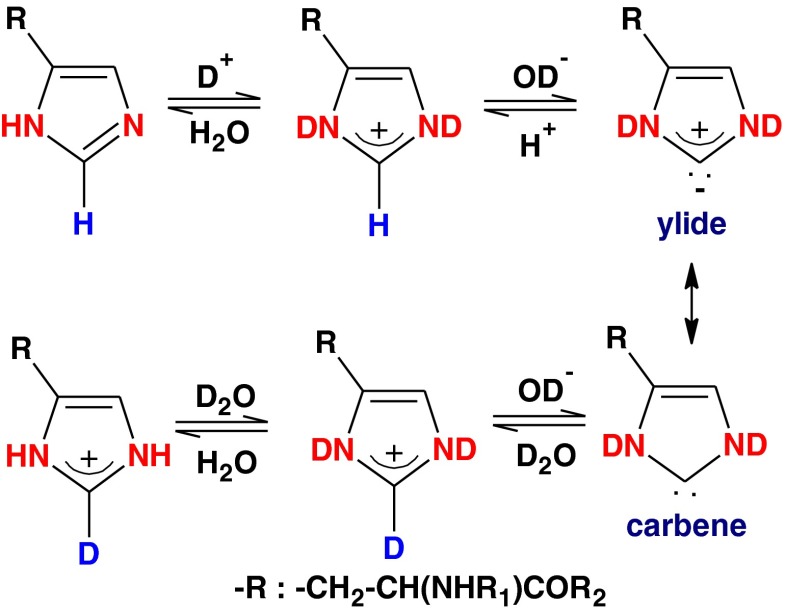
Mechanism of HDX exchange in histidine

It is known that the imidazole C_2_ proton (also denoted the C_ε1_ proton) can be exchanged with a deuteron by incubating the imidazole in deuterium oxide (D_2_O) [19]. The half-life of the hydrogen–deuterium exchange (HDX) reaction in an unmodified histidine residue is on the order of 2 days [20], which is about 4 × 10^5^ times slower than that of the HDX reaction of an unprotected amide proton [21]. The HDX reaction of the imidazole ring presents pseudo-first-order kinetics [22, 23]. The rate-determining step is the formation of an ylide or a carbene intermediate via the abstraction of the C_ε1_ proton from the cationic imidazolium by deuteroxyl ions (Fig. 2) [24]. The rate profile of the HDX reaction as a function of pD is a sigmoidal curve that rises at acidic pD before plateauing out at alkaline pD [20]. This profile yields two useful parameters that indicate the microenvironment of the imidazole group in a peptide or a protein. The first is the p*K*_a_ of the imidazole NH group, which converges with the inflection point of the curve described above. The reason for this is the dependency of the HDX reaction rate on the concentrations of the cationic imidazolium and deuteroxyl ions. The second parameter is the maximum pseudo-first-order rate constant* k* ^max^, which is derived from the upper plateau of the sigmoidal curve.

The experimentally obtained p*K*_a_ values of imidazole NH groups are indicators of the electrostatic microenvironments of histidine imidazole groups in the proteins [20]. The reason for this is the sensitivity of the p*K*_a_ value to adjacent charged groups. On the other hand, the accessibility of an imidazole group to the bulk solvent has a significant effect on its HDX rate [25]. As a result,* k* ^max^ values at individual imidazole groups are used to gauge the solvent accessibility of His. A recent report indicates that metal ion complexation also has a significant effect on the DHX reaction of the C_2_ proton in the imidazole ring of histidine [26]. This observation allows the metal-binding sites in polypeptides to be determined through a combination of HDX and mass spectrometry.

Early kinetic data on HDX in the imidazole moiety clearly demonstrated that the rate constant of this reaction is correlated with the charge on the imidazole ring. Since phosphoramidate introduces a negative charge in the proximity of the C_2_ atom, we expected that phosphorylation of the His residue would influence the rate of isotope exchange. If this assumption is true, HDX at the histidine side chain may be a simple alternative method of unambiguously monitoring for histidine phosphorylation in polypeptides and proteins. Therefore, the aim of the study reported in the present paper was to determine the influence of the peptide sequence and the phosphorylation of a histidine moiety on the kinetics of HDX at the C_2_ atom of the imidazole ring in that histidine.

## Materials and methods

### Reagents

Reagents [(NH_4_)_2_CO_3_; NH_4_HCO_3_; CH_3_COONH_4_; CH_3_COOH; NH_3_ solution in H_2_O; D_2_O; TCTU; DIEA; TIS] and solvents (MeCN, DMF; DCM; MeOH) were purchased from Sigma–Aldrich (St. Louis, MO, USA) and used without further purification. SepPak C18 Plus Light cartridges (Waters Corp., Milford, MA, USA) were used for desalting (parameters: 130 mg sorbent per cartridge, 55–105 μm particle size). Formic acid (99 %) was purchased from Merck (Darmstadt, Germany).

### Solid-phase peptide synthesis

Ten peptides (H-Ala-Ala-Arg-His-Ala-Phe-OH; H-Ala-Ala-Asp-His-Ala-Phe-OH; H-Ala-Arg-Ala-His-Ala-Phe-OH; H-Ala-Asp-Ala-His-Ala-Phe-OH; H-Arg-Ala-Ala-His-Ala-Phe-OH, H-Asp-Ala-Ala-His-Ala-Phe-OH; H-Ala-Ala-Ala-His-Ala-Phe-OH; H-βAsp-Arg-Val-Tyr-Ile-His-Pro-Phe-OH; H-Asn-His-Phe-Trp-Lys-Thr-His-Thr-NH_2_, H-His-Tyr-Ile-Gln-Asn-His-Pro-Leu-Gly-NH_2_) were synthesized on a solid support (Wang resin, loading 0.77 mM/g) using the standard Fmoc strategy. The coupling reagent was TCTU with DIEA in DMF. The degree of conversion was checked by performing the Kaiser test. The peptides were cleaved from the resin using a mixture of TFA, H_2_O, and TIS (95:2.5:2.5, v:v:v) and purified by preparative HPLC. Analytical data for all the peptides obtained are given in the “Electronic supplementary material,” (ESM).

### HDX protocols

#### Procedure I for the HDX reaction

A series of buffer solutions containing volatile salts, acids, and bases [(NH_4_)_2_CO_3_, NH_4_HCO_3_, CH_3_COONH_4_, CH_3_COOH, NH_3_ solution in H_2_O] in D_2_O were prepared. The concentration of each solution was 10 mM; the solutions differed only in their pH values. Each peptide (2.2 mg) was dissolved in 90 μl D_2_O. A 10-μl aliquot of this solution was transferred to an Eppendorf tube and mixed with a corresponding buffer solution (115 μl). Sealed Eppendorf tubes were thermostated at 40 °C. After a given reaction time, the products were analyzed to determine the HD exchange level. A 10-μl aliquot was collected and mixed with 1 μl HCOOH. According to data in the literature as well as our experiments, acidification completely stops HDX from occurring in the imidazole ring. The sample was then lyophilized to remove D_2_O. The lyophilisate was dissolved in a solvent used for ESI-MS measurements (H_2_O:MeCN:HCOOH 50:50:0.1; v:v:v) and incubated at room temperature for 1 h before performing the MS measurements. During this procedure, amide deuterons and other labile deuterons were replaced by protons, while deuteration at the C_2_ carbon atom of the imidazole ring was preserved.

#### Procedure II for the DHX reaction

- **Deuteration at C**_**2**_ **of His imidazole**
  Two milligrams of each unmodified peptide were dissolved in 0.5 ml 10 mM (NH_4_)_2_CO_3_ in D_2_O in a sealed Eppendorf tube. The solutions were thermostated at 60 °C for 96 h. After that time, the reaction was terminated by adding 50 μl HCOOH and the samples were lyophilized. To check the level of deuteration, the peptides were dissolved in 200 μl of the solvent used for ESI-MS (H_2_O:MeCN:HCOOH 50:50:0.1) and incubated for 1 h before the measurement. The spectra obtained reveal that, in every sample, only one proton was replaced with a deuteron. Deuterated samples were lyophilized and stored for further analysis.
- **A kinetic reaction analysis**
  A series of buffer solutions in H_2_O-containing volatile salts, acids, and bases [(NH_4_)_2_CO_3_, NH_4_HCO_3_, CH_3_COONH_4_, CH_3_COOH, NH_3_ solution in H_2_O] were prepared. The concentration of each solution was 50 mM; the solutions differed only in their pH values. A deuterated unmodified peptide from the previous experiment (a) was mixed with 80 μl H_2_O and 10 μl of this solution were placed in an Eppendorf tube. In the next step, 115 μl of the buffer solution were added. The Eppendorf tubes were thermostated at 40 °C. After a given reaction time, the products were analyzed. A 10-μl aliquot of a sample was collected and mixed with 100 μl of the H_2_O:MeCN:HCOOH (50:50:0.1) mixture and ESI-MS measurements were performed immediately.
- **Procedure for peptides with pHis**
  For the peptides designed for phosphorylation, the deuteration reaction was carried out at pH 9. The same procedure was applied as in experiment (a). After 96 h, the phosphorylating reagent potassium phosphoramidate (KHPO_3_NH_2_, 50 mg) was added to a deuterated peptide (1 mg) [27, 28]. Samples of both deuterated and phosphorylated peptides were desalted on reversed-phase columns (SepPak) and evaporated to dryness under a stream of nitrogen. The samples were reconstituted with a buffer of a defined pH and incubated at 40 °C. After a given reaction time, the products were analyzed. A 10-μl aliquot of a sample was collected and mixed with 100 μl of the H_2_O:MeCN:HCOOH (50:50:0.1) mixture and ESI-MS measurements were performed immediately.

#### HPLC

Analytical high-performance liquid chromatography (HPLC) was carried out on a Thermo Separation (Waltham, MA, USA) HPLC system using a Vydac RP C18 column (4.6 × 250 mm) with ultraviolet (UV) detection at 220 nm. The flow rate was 1 ml/min and the gradient was 0–80 % B in A for 40 min, where A was water containing 0.1 % TFA and B was acetonitrile containing 0.1 % TFA.

Preparative high-performance liquid chromatography (HPLC) was carried out on a Varian (Palo Alto, CA, USA) ProStar HPLC system using a TOSOH Bioscience (Minato, Japan) TSKgel RP ODS 120 T column (21.5 × 300 mm) with ultraviolet (UV) detection at 280 and 220 nm. The flow rate was 7 ml/min and the gradient parameters varied for different peptides. The solvents were: A, water containing 0.1 % TFA; B, acetonitrile:water (4:1, v:v) containing 0.1 % TFA.

#### MS and MS/MS experiments

All MS experiments were performed on an Apex-Qe Ultra 7 T instrument (Bruker Daltonics, Bremen, Germany) equipped with an electrospray ionization (ESI) source. The instrument was operated in the positive ion mode and calibrated with the Tunemix mixture (Bruker Daltonics). The samples for MS and MS/MS experiments were dissolved in an acetonitrile/water/formic acid mixture (50:50:0.1, v/v/v). The obtained mass spectra were analyzed using data analysis software from Bruker Daltonics. The mass accuracy was better than 5 ppm. The instrument parameters were as follows: scan range, 300–2,500 *m*/*z*, drying gas, nitrogen; temperature of drying gas, 200 °C; potential between the spray needle and the orifice, 4.5 kV; source accumulation time, 0.5 s; ion accumulation time, 0.5 s. In the MS/MS mode, the precursor ions were selected in the quadrupole collision cell and subsequently fragmented in the hexapole collision cell using argon as collision gas. The collision energy in the hexapole collision cell was set at 12–12.5 eV. The obtained fragment ions were directed to the ICR mass analyzer and registered as MS/MS spectra.

## Results and discussion

To analyze the effect of the model peptide sequence on the isotope exchange kinetics, we synthesized a series of seven peptides, each containing a His residue. Three of them were basic (one Arg residue), three were acidic (one Asp residue), and one peptide was neutral (it only contained Ala and Phe residues besides His). The pseudo-first-order rate constant (*k*) of the isotope exchange reaction was determined for each peptide and each pH/pD value. The* k* value was determined by the least squares method from the equation ln*Y* = *kt*, where* t* is the isotope exchange time and* Y* is a function of the relative abundances of isotope peaks in MS spectra [24]:

$$ Y=\frac{I_{\mathrm{M}+1}(t)}{I_{\mathrm{M}}(t)}-\frac{I_{\mathrm{M}+1}(0)}{I_{\mathrm{M}}(0)}+1. $$

Only the M and M+1 peaks were used for these calculations. The intensities of the M and M+1 peaks at time* t* = 0 were denoted* I*_M_(0) and* I*_M+1_(0), respectively. Similarly, the intensities of the M and M+1 peaks at time* t* were denoted* I*_M_(*t*) and* I*_M+1_(*t*).

Based on the dependence of the calculated* k* values on the pH, the parameters p*K*_a_ and* k* ^max^ were determined by fitting the experimental values to the equation

$$ \mathrm{p}{K}_{\mathrm{a}}=\mathrm{p}\mathrm{H}+ \log \left(\frac{k^{\max }-k}{k}\right). $$

### HDX for the unmodified peptides: procedure I

The HDX experiment on the C_2_ proton of imidazole was performed for nonphosphorylated peptides only. This reaction took place in buffer containing D_2_O at 40 °C, and after a defined time it was quenched by acidifying the solution. After the time needed to allow the back exchange of fast-exchanging deuterons (approx. 1 h) had elapsed, the peptides were subjected to MS analysis.

**Fig. 3:**
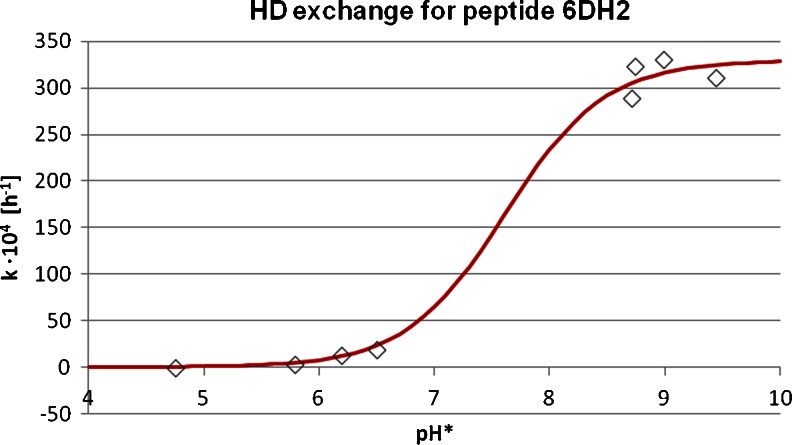
Dependence of the HDX rate constant on pH for the peptide H-Asp-Ala-Ala-His-Ala-Phe-OH (6DH2)

Figure 3 shows the dependence of the* k* value on the pH for compound 6DH2. The dependencies of* k* on pH for other peptides are given in the ESM. These data enabled the determination of p*K*_a_ and* k* ^max^. The values obtained are collected in Table 1. The results suggest that a neighboring guanidine group lowers the p*K*_a_ of the imidazole group (as in the cases of 1RH0, 3RH1, and 5RH2), whereas a neighboring carboxyl group increases the p*K*_a_ (as in the cases of 2DH0 and 4DH1). This effect is consistent with the well-established fact that neighboring positively charged groups lower the p*K*_a_ of the imidazole moiety while neighboring negatively charged groups increase it. The* k* values for all of the studied peptides are similar (250–323 × 10^−4^; standard deviation for the whole series: 8.4 %), while the* k* value does not change in a regular manner and does not correlate well with the peptide sequence. This fact may be explained by the flexibility of linear peptides and the relatively large distances between the imidazole ring and the charged side chains.

**Table 1:** Values of p*K*_a_ and* k* ^max^ for HDX and DHX reactions of the analyzed native peptides

Peptide name	Peptide sequence	p*K*_a_ value for DHX	*k*_max_ value for DHX (h^−1^)	p*K*_a_ value for HDX	*k*_max_ value for HDX (h^−1^)
1RH0	H-Ala-Ala-Arg-His-Ala-Phe-OH	7.01	250×10^−4^	7.23	234×10^−4^
2DH0	H-Ala-Ala-Asp-His-Ala-Phe-OH	7.58	284×10^−4^	7.72	331×10^−4^
3RH1	H-Ala-Arg-Ala-His-Ala-Phe-OH	7.37	263×10^−4^	6.71	230×10^−4^
4DH1	H-Ala-Asp-Ala-His-Ala-Phe-OH	7.20	279×10^−4^	7.63	331×10^−4^
5RH2	H-Arg-Ala-Ala-His-Ala-Phe-OH	7.67	291×10^−4^	7.23	258×10^−4^
6DH2	H-Asp-Ala-Ala-His-Ala-Phe-OH	7.59	255×10^−4^	7.49	330×10^−4^
70H0	H-Ala-Ala-Ala-His-Ala-Phe-OH	7.61	323×10^−4^	7.56	270×10^−4^

Direct HDX reaction monitoring is rather time-consuming and inconvenient. Before each measurement, D_2_O has to be removed from the sample by lyophilization, and the back-exchange of amide deuterons requires additional time (approx. 1 h). The reaction mixture containing D_2_O is susceptible to contamination with atmospheric water. In addition, the consumption of D_2_O in this approach is relatively high. To simplify measurements, we decided to develop an alternative procedure based on deuterium–hydrogen exchange in subsequent studies.

### DHX in the unmodified peptides: procedure II

The second analytical method is based on the DHX reaction. This procedure is faster and _decrease in the D_2_O volume used reduces costs. In this method the deuteration is conducted only once for each peptide at the beginning of the process. Moreover, removal of D_2_O from the sample and back-exchange of amide deuterons are performed only once after the deuteration of a peptide. This significantly reduces the time required for the analysis. The water contamination and the error caused by the use of nondeuterated salt in buffers do not constitute significant problems.

As in the previous procedure, a plot of the reaction rate versus pH (created using a set of buffer solutions with only one peptide) allows the p*K*_a_ and* k* ^max^ parameters to be determined. The data obtained are also collected in Table 1.

Again, although the chemical environment of the imidazole ring varies, the* k* ^max^ values are found to be relatively close to each other. The highest value is 331 × 10^−4^ and the lowest 230 × 10^−4^, while the standard deviation for the whole series is 15.1 %. Changes in the maximum rate constant are not regular, and its correlation with the peptide sequence is difficult to determine. Data obtained for HDX and DHX reactions indicate that the kinetics of the isotope exchange of imidazole C_2_ protons are similar for a wide range of peptides and the* k* ^max^ value is not sensitive to changes in the sequence of the peptide.

### CID fragmentation of the unmodified peptides after HD exchange

**Fig. 4:**
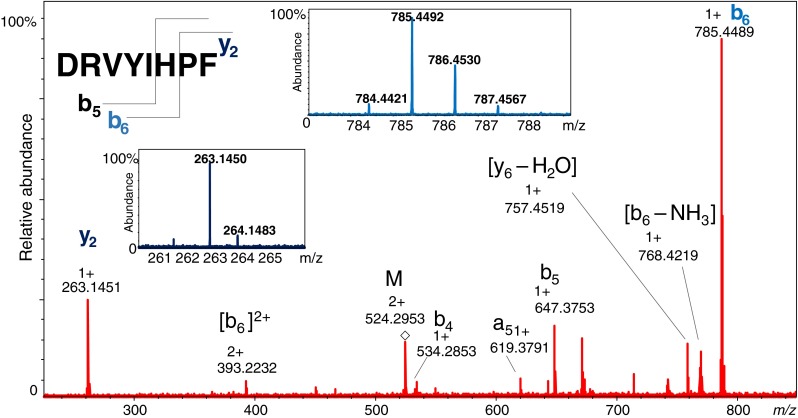
MS/MS spectrum of H-βAsp-Arg-Val-Tyr-Ile-His-Pro-Phe-OH. The isotope pattern for the b_6_ ion has been expanded

CID experiments were conducted for three peptides with the His residue. Fragmentation of the peptide H-βAsp-Arg-Val-Tyr-Ile-His-Pro-Phe-OH confirmed that HDX occurs at the imidazole ring of the His residue. The y and b fragment ions (which contain His residues) have characteristic isotope distributions in the MS/MS spectrum that indicate the incorporation of deuterium (Fig. 4). The absence of deuterium in fragments which do not contain the His residue suggests a lack of scrambling during MS/MS experiments. As a result, the CID technique for deuterated multihistidine peptides allows the His involved in the HDX reaction to be determined. It is worth noting that we observed the opposite effect in our recent work on the slow HDX of protons attached to the α-carbon in sarcosine-containing peptides: unexpected hydrogen scrambling during the collision-induced dissociation [29].

### DH exchange in the phosphorylated peptides: procedure II

**Fig. 5:**
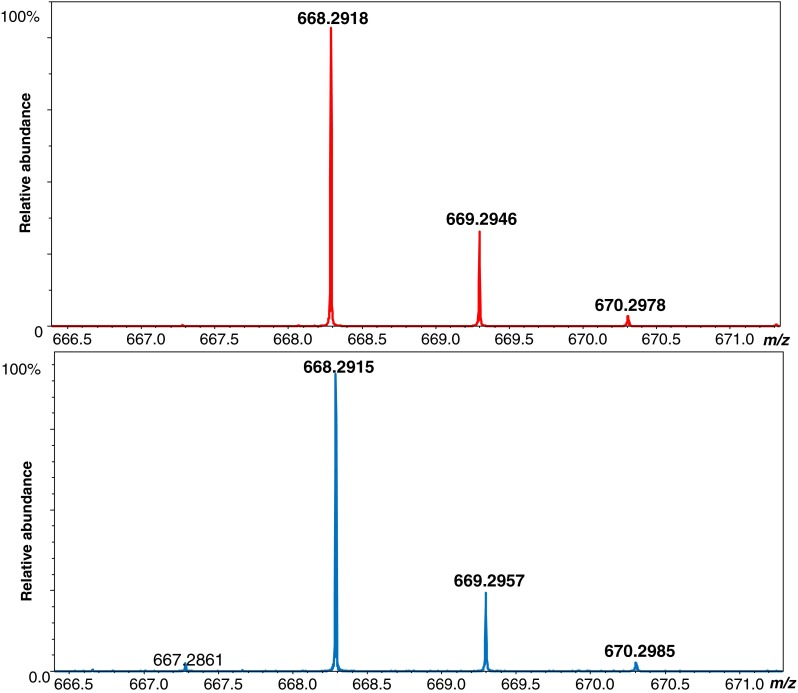
MS spectra for the monodeuterated peptide H-Ala-Ala-Ala-pHis-Ala-Phe-OH containing phosphohistidine before (*upper panel*) and after (*lower panel*) deuterium–hydrogen exchange. The spectra clearly demonstrate that DHX proceeds at a slow rate for peptides with pHis

Isotope exchange measurements of phosphorylated peptides must be conducted in an alkaline environment because this modification is unstable at acidic pH. Procedure II, based on the DHX reaction, was applied to analyze this process. The* k* values obtained for all phosphorylated peptides measured at four pH values are collected in Table 2. According to those results, the isotope exchange reaction in pHis occurs to only a negligible extent. Reaction rates are on the order of ~2.4–5.4 × 10^4^ h^−1^, which is significantly smaller (by two orders) than the reaction rates for native peptides. Because of the low values of the measured constants (which are essentially within the experimental error bars), p*K*_a_ and* k* ^max^ could not be determined. MS spectra obtained before and after isotope exchange in the phosphorylated peptide 70H0—as presented in Fig. 5—clearly demonstrate that the DHX for peptides with pHis proceeds at a very slow rate.

**Table 2:** Kinetic rate constants* k* for DHX reactions of the analyzed phosphorylated peptides at four different pH values

Peptide name	pH 7.9	pH 8.2	pH 8.7	pH 9.0
	*k* value for DHX for phosphorylated peptides (*k*×10^−4^ h^−1^)			
1RH0	4.2	3.7	2.8	2.5
2DH0	3.4	4.4	4.3	4.6
3RH1	3.3	3.9	3.3	2.4
4DH1	3.8	3.2	3.6	4.6
5RH2	5.4	5.3	2.4	3.1
6DH2	3.2	3.2	4.2	3.7
70H0	3.7	3.2	3.2	3.2

It is generally difficult to detect phosphohistidine in modified proteins using standard proteomic protocols because of the instability of the phosphate–nitrogen bond under acidic conditions [30]. Therefore, the search for various differences between the native and the phosphorylated histidine residue is important, as these differences may be used to analyze this type of phosphorylation.

One such method, implemented by Sun et al. [31], involves the use of iodine labeling and tandem mass spectrometry (MS/MS) to study the sites of histidine phosphorylation. The histidine residues in peptides undergo iodination under mild conditions while pHis remains unmodified. Acid treatment removes all of the phosphoroamidate groups. In contrast, iodinated His is stable under these conditions. MS/MS methods can be used to identify and distinguish site-specific modifications. Thus, the iodinated residues correspond to the previously unphosphorylated His while the free His residues correspond to the previously phosphorylated ones. This method is semi‐quantitative, enabling relative abundances to be determined with an accuracy of approximately ±20 %. The drawback of this method is the lack of specificity of iodination, because this modification occurs at both histidine and tyrosine residues. Moreover, tyrosine is more easily iodinated than histidine.

The method presented in the present work is analogous to that mentioned above. Our results clearly demonstrate that phosphorylation of imidazole inhibits both the DHX and the HDX reactions. Decreases in reaction rate constants can be easily detected by MS measurements, enabling the identification of pHis in peptides and proteins. The addition of an acid to the sample of an enzymatic digest causes rapid dephosphorylation of pHis residues and stops isotope exchange in the imidazole ring. As a result, both phosphorylated (pHis) and unphosphorylated (His) histidine—detected as free His moieties—may be distinguished on the basis of their isotopic profiles. In peptides phosphorylated at the His moiety, limited incorporation of deuterium atoms is expected, while previously unmodified His residues should undergo HDX at the C2 atom of the imidazole ring.

## Conclusions

Phosphohistidine is an example of a phosphoamino acid that is labile under acidic conditions. The low stabilities of such compounds make it challenging to analyze them. According to our results, phosphorylation dramatically slows the rate of DHX. We demonstrated that the* k* values measured for a peptide with pHis differ by about two orders of magnitude from the reaction rates measured for an unmodified peptide. On the other hand, the effect of the sequence on the HDX kinetics is negligible. Consequently, this rate constant provides precise information about the presence of phosphohistidines in peptides. Standard LC methods performed under acidic conditions as well as tandem mass spectrometry can be implemented in future analyses of phosphorylation sites. Moreover, CID experiments performed after initial HDX demonstrated that hydrogen scrambling does not take place, meaning that the site at which the deuterium is incorporated can be determined (e.g., in multihistidine peptides).

## Electronic supplementary material

Below is the link to the electronic supplementary material.

ESM 1 (PDF 547 KB)
