# Supplementary material for: Hydrogen–deuterium exchange in imidazole as a tool for studying histidine phosphorylation
Source: Anal Bioanal Chem. 2014 Oct 30;406(30):8013–20. doi: 10.1007/s00216-014-8218-5 (PMC4244536; doi:10.1007/s00216-014-8218-5)
Supplement: Supplementary file 1 — (PDF 547 KB) [file 216_2014_8218_MOESM1_ESM.pdf]

Analytical and Bioanalytical Chemistry

Electronic Supplementary Material

Hydrogen-deuterium exchange in imidazole as a tool for studying histidine phosphorylation

Małgorzata Cebo, Martyna Kielmas, Justyna Adamczyk, Marek Cebrat, Zbigniew Szewczuk, Piotr Stefanowicz*

Table S1. The comparison of calculated and experimental masses for $[MH]^+$ ions of model peptides and their monodeuterated analogues

Peptide name	Peptide sequence	Molecular formula	Native peptide Found mass [Da]	Native peptide Calculated mass [Da]	Deuterated peptide Calculated mass [Da]	Deuterated peptide Found mass[Da]
**1RH0**	H-**Ala**-**Ala**-**Arg**-**His**-**Ala**-**Phe**-OH	$C_{30}H_{45}N_{11}O_7$	672.3764	672.3582	673.3639	673.3893
**2DH0**	H-**Ala**-**Ala**-**Asp**-**His**-**Ala**-**Phe**-OH	$C_{28}H_{38}N_8O_9$	631.2815	631.2835	632.2897	632.3107
**3RH1**	H-**Ala**-**Arg**-**Ala**-**His**-**Ala**-**Phe**-OH	$C_{30}H_{45}N_{11}O_7$	672.3822	672.3582	673.3639	673.3882
**4DH1**	H-**Ala**-**Asp**-**Ala**-**His**-**Ala**-**Phe**-OH	$C_{28}H_{38}N_8O_9$	631.2805	631.2835	632.2897	632.3134
**5RH2**	H-**Arg**-**Ala**-**Ala**-**His**-**Ala**-**Phe**-OH	$C_{30}H_{45}N_{11}O_7$	672.3822	672.3582	673.3639	673.3891
**6DH2**	H-**Asp**-**Ala**-**Ala**-**His**-**Ala**-**Phe**-OH	$C_{28}H_{38}N_8O_9$	631.3058	631.2835	632.2897	632.3114
**70H0**	H-**Ala**-**Ala**-**Ala**-**His**-**Ala**-**Phe**-OH	$C_{27}H_{38}N_8O_7$	587.3133	587.2936	588.2999	588.3195

Table S2. The selected fragment ions for model peptides

Peptide name	Peptide sequence	Ion fragments	Found mass [Da]	Calculated mass [Da]
**1RH0**	H-**Ala**-**Ala**-**Arg**-**His**-**Ala**-**Phe**-OH	b4	436.2427	436.2421
		y3	374.1855	374.1829
**2DH0**	H-**Ala**-**Ala**-**Asp**-**His**-**Ala**-**Phe**-OH	b5	466.2195	466.2051
		y4	489.2253	489.2098
**3RH1**	H-**Ala**-**Arg**-**Ala**-**His**-**Ala**-**Phe**-OH	a5	479.2995	479.2843
		b4	436.3552	436.2421
**4DH1**	H-**Ala**-**Asp**-**Ala**-**His**-**Ala**-**Phe**-OH	b6	613.2953	613.2735
		y4	445.2333	445.2200
**5RH2**	H-**Arg**-**Ala**-**Ala**-**His**-**Ala**-**Phe**-OH	a5	479.2996	479.2843
		b4	436.2552	436.2421
**6DH2**	H-**Asp**-**Ala**-**Ala**-**His**-**Ala**-**Phe**-OH	b4	395.1675	395.1680
		y3	374.1846	374.1829
**70H0**	H-**Ala**-**Ala**-**Ala**-**His**-**Ala**-**Phe**-OH	b5	422.2274	422.2152
		y4	445.2334	445.2200

Table S3. The comparison of calculated and experimental masses for $[MH]^+$ ions of monodeuterated analogues of phosphorylated peptides

Peptide name	Peptide sequence	Peptide with pHis Calculated mass	Deuterated peptide with pHis Calculated mass	Deuterated peptide with pHis Found mass
**1RH0**	H-**Ala-Ala-Arg-His-Ala-Phe**-OH	752.3239	753.3302	753.3595
**2DH0**	H-**Ala-Ala-Asp-His-Ala-Phe**-OH	711.2498	712.2561	712.2833
**3RH1**	H-**Ala-Arg-Ala-His-Ala-Phe**-OH	752.3239	753.3302	753.3604
**4DH1**	H-**Ala-Asp-Ala-His-Ala-Phe**-OH	711.2498	712.2561	712.2844
**5RH2**	H-**Arg-Ala-Ala-His-Ala-Phe**-OH	752.3239	753.3302	753.3606
**6DH2**	H-**Asp-Ala-Ala-His-Ala-Phe**-OH	711.2498	712.2561	712.2826
**70H0**	H-**Ala-Ala-Ala-His-Ala-Phe**-OH	667.2600	668.2662	668.2905

Table S4. CID fragmentation pattern for peptide **DRVYJHPF**

Fragment	Molecular formula	Calculated m/z	Experimental m/z
y_2^+	$C_{14}H_{19}N_2O_3$	263.1239	263.1451
$[b_6]^{2+}$	$C_{36}H_{55}N_{11}O_9$	393.2131	393.2232
b_4^+	$C_{24}H_{36}H_7O_7$	534.2676	534.2853
a_5^+	$C_{29}H_{47}N_8O_7$	619.3568	619.3791
b_5^+	$C_{30}H_{47}N_8O_8$	647.3517	647.3753
$[y_6\text{-}H_2O]^+$	$C_{40}H_{53}N_8O_7$	757.4037	757.4519
$[b_6\text{-}NH_3]^+$	$C_{36}H_{50}DN_{10}O_9$	768.4005	768.4219
b_6^+	$C_{36}H_{53}DN_{11}O_9$	785.4169	785.4489

Table S5. CID fragmentation pattern for peptide NHFWKTHT

Fragment	Molecular formula	Calculated m/z	Experimental m/z
$[b_2\text{-}NH_3]^+$	$C_{10}H_{11}N_4O_3$	235.0863	235.0822
b_2^+	$C_{10}H_{14}N_5O_3$	252.1097	252.1088
$[y_3\text{-}NH_3]^+$	$C_{14}H_{19}DN_5O_5$	341.1715	341.1840
y_3^+	$C_{14}H_{22}DN_6O_5$	358.1949	358.1884
b_3^+	$C_{19}H_{23}N_6O_4$	399.1781	399.1783
$[b_4\text{-}NH_3]^+$	$C_{30}H_{30}N_7O_5$	568.2340	568.2369
b_4^+	$C_{30}H_{33}N_8O_5$	585.2574	585.2603
$[z_5\text{-}H_2O]^+$	$C_{31}H_{41}DN_9O_6$	637.3322	637.3296
$[y_5\text{-}H_2O]^+$	$C_{31}H_{42}DN_{10}O_6$	654.3587	654.3570
y_5^+	$C_{31}H_{44}DN_{10}O_7$	672.3692	672.3675
b_5^+	$C_{36}H_{45}N_{10}O_6$	713.3524	713.3581
$[z_6\text{-}H_2O]^+$	$C_{40}H_{50}DN_{10}O_7$	784.4006	784.4070
$[y_6\text{-}H_2O]^+$	$C_{40}H_{51}DN_{11}O_7$	801.4271	801.4344
y_6^+	$C_{40}H_{53}DN_{11}O_8$	819.4376	819.4451

Table S6. CID fragmentation pattern for peptide **HYIQNHPLG**

Fragment	Molecular formula	Calculated m/z	Experimental m/z
y_3^+	$C_{13}H_{25}N_4O_3$	285.1770	295.1994
b_2^+	$C_{15}H_{16}DN_4O_3$	302.1364	302.1434
$[b_6]^{2+}$	$C_{36}H_{49}DN_{12}O_9$	398.1982	398.1910
b_3^+	$C_{21}H_{27}DN_5O_4$	415.2204	415.2261
$[a_8]^{2+}$	$C_{46}H_{67}DN_{14}O_{10}$	489.2692	489.2126
$[b_8]^{2+}$	$C_{47}H_{67}DN_{14}O_{11}$	503.2666	503.2787
$[b_5\text{-}H_2O]^+$	$C_{30}H_{39}DN_9O_8$	639.3114	639.3002
b_5^+	$C_{30}H_{41}DN_9O_8$	657.3219	657.3404
$[b_6\text{-}CO]^+$	$C_{35}H_{48}DN_{12}O_8$	766.3859	766.3432
y_7^+	$C_{34}H_{55}N_{12}O_9$	777.4215	777.4690
b_6^+	$C_{36}H_{48}DN_{12}O_9$	794.4140	794.4104
z_8^+	$C_{43}H_{62}DN_{12}O_{11}$	923.5039	923.5138
y_8^+	$C_{43}H_{64}DN_{13}O_{11}$	940.5148	940.5438

IMAGES

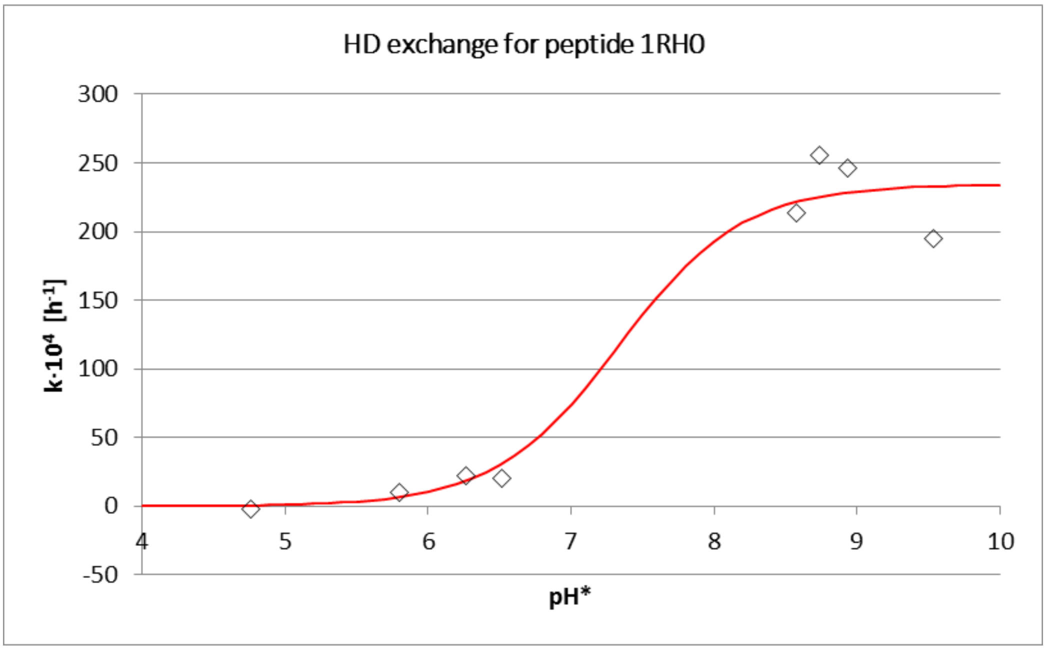

Fig. S1. The dependence of HDX rate constant on pH for peptide 1RH0

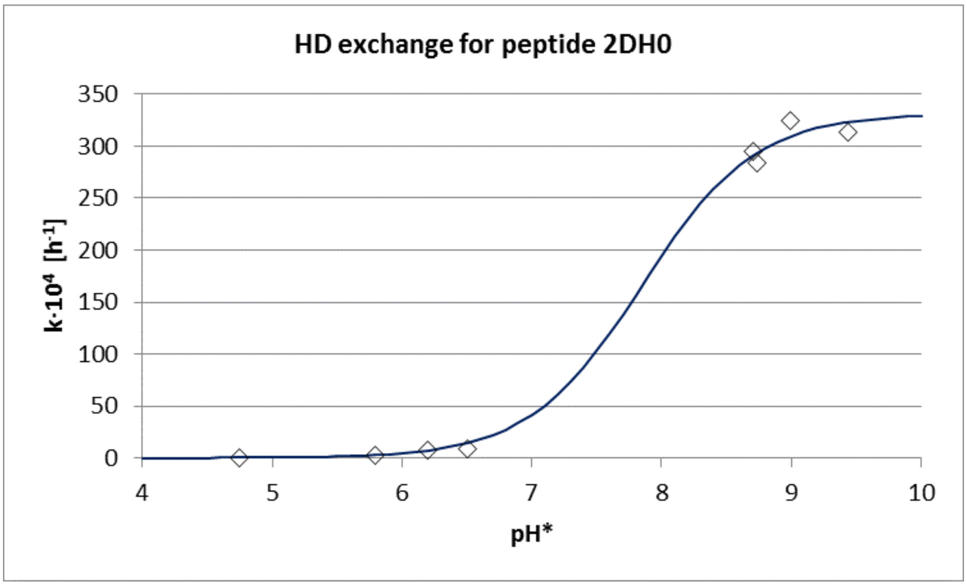

Fig. S2. The dependence of HDX rate constant on pH for peptide 2DH0

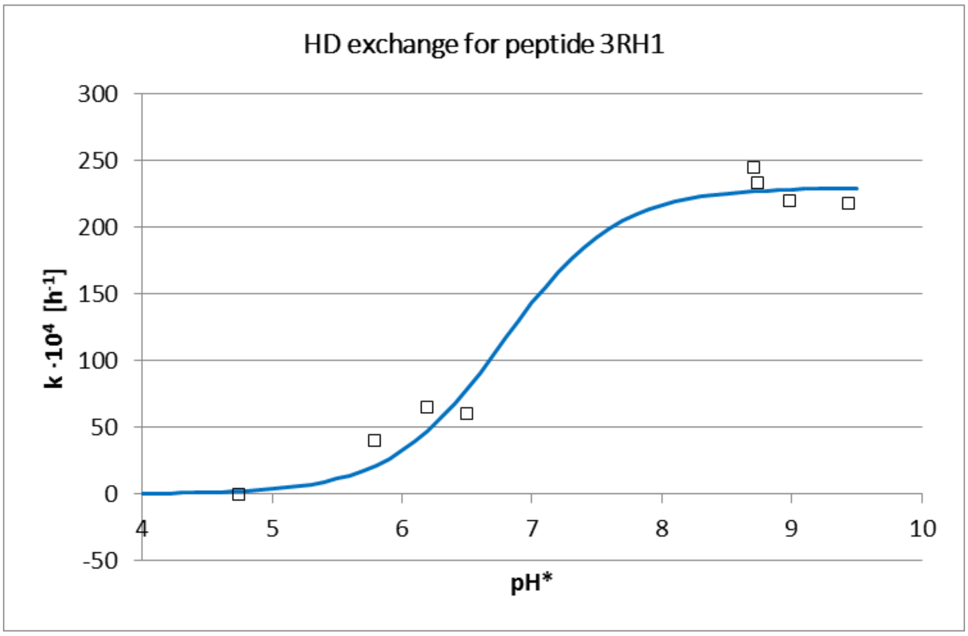

Fig. S3. The dependence of HDX rate constant on pH for peptide 3RH1

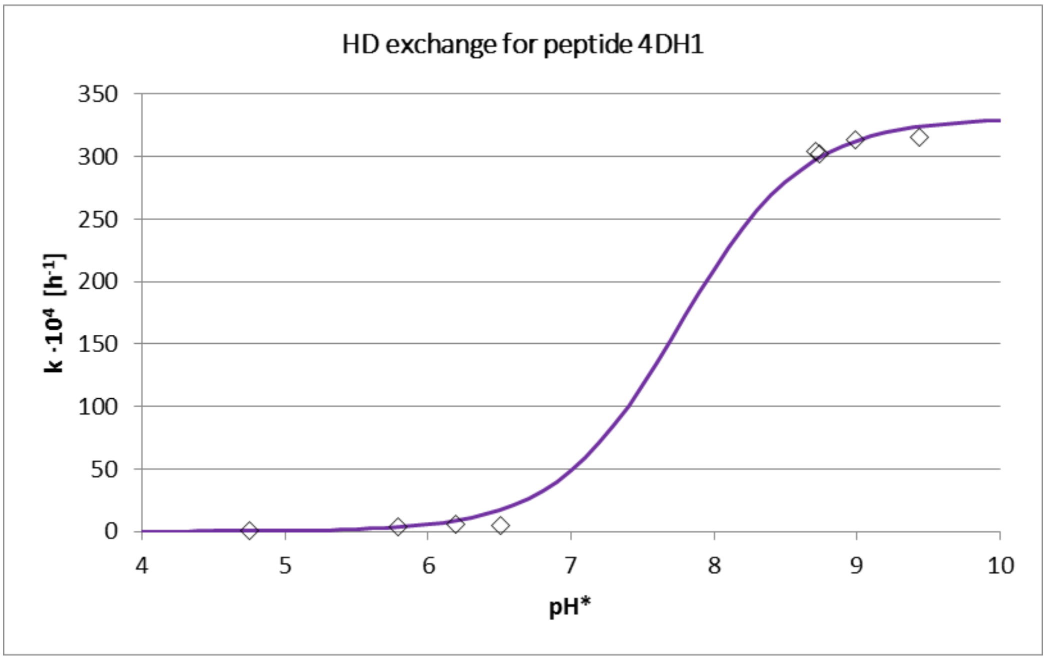

Fig. S4. The dependence of HDX rate constant on pH for peptide 4DH1

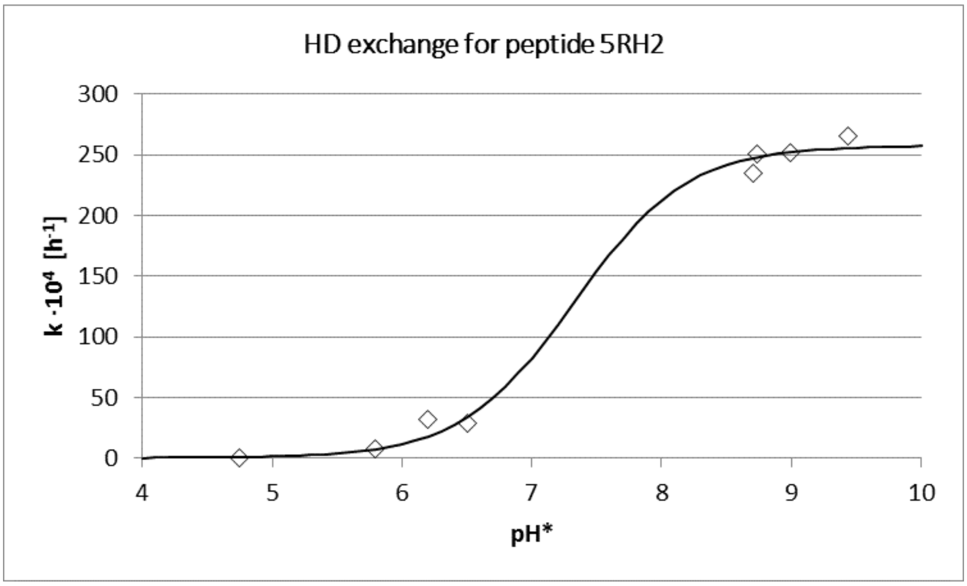

Fig. S5. The dependence of HDX rate constant on pH for peptide 5RH2

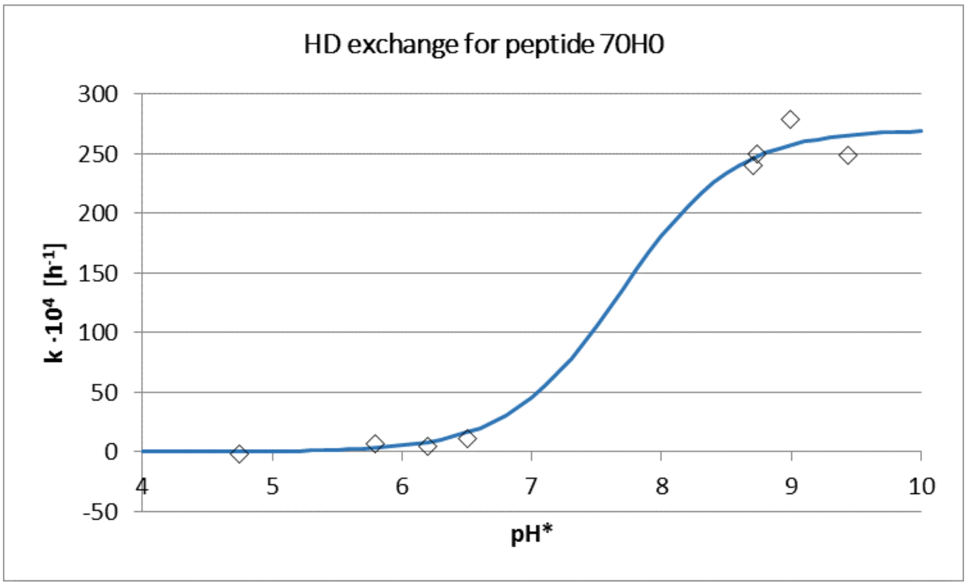

Fig. S6. The dependence of HDX rate constant on pH for peptide 70H0

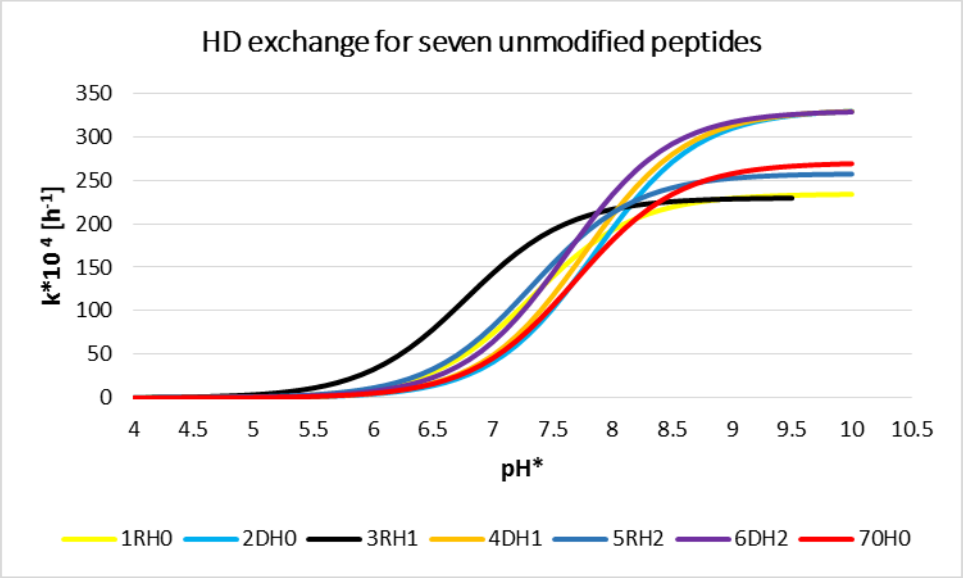

Fig. S7. The dependence of HDX rate constant on pH for all seven unmodified peptide
